# Investigating a modified Yiqi Huoxue formula for chronic glomerulonephritis: Effects on clinical efficacy, renal function, and inflammation

**DOI:** 10.1097/MD.0000000000047680

**Published:** 2026-02-20

**Authors:** Xi Liu, Jing Sun

**Affiliations:** a Department of Traditional Chinese Medicine, Beijing Jingshun Hospital of TCM, Beijing, China; b Internal Medicine of Traditional Chinese Medicine, Beijing Xuanwu Traditional Chinese Medicine Hospital, Beijing, China.

**Keywords:** glomerulonephritis, inflammatory markers, modified Yiqi Huoxue formula, renal function

## Abstract

This study aimed to evaluate the clinical efficacy of a modified Yiqi Huoxue formula in patients with chronic glomerulonephritis (CGN), with a specific focus on its impact on renal function and inflammatory markers. In this Retrospective study, patients diagnosed with CGN and the information collected in the outpatient medical record between April and August 2023 were allocated to either an observation group or a control group. The control group (n = 45) received conventional basic treatment, whereas the observation group (n = 45) was administered the modified Yiqi Huoxue formula alongside conventional treatment for a duration of 8 weeks. Primary outcomes included clinical efficacy rates, while secondary outcomes encompassed measurements of renal function indices (serum creatinine [Scr], blood urea nitrogen [BUN], 24-hour urinary protein quantitation [24hUPQ]), lipid profiles (triglycerides [TG], total cholesterol, low-density lipoprotein cholesterol [LDL-C]), and inflammatory markers (interleukin-6 [IL-6], high-sensitivity C-reactive protein [hs-CRP]). A total of 90 patients were enrolled. The observation group exhibited a significantly higher clinical effectiveness rate of 86.67% compared to 66.67% in the control group (*P* <.05). After the 8-week intervention, posttreatment levels of Scr, BUN, and 24hUPQ were significantly lower in the observation group than in the controls (*P* <.05). Furthermore, significant reductions were also observed in the observation group for TG, total cholesterol, LDL-C, IL-6, and hs-CRP levels (*P* <.05). Multivariate logistic regression analysis identified reductions in Scr, BUN, 24hUPQ, IL-6, and hs-CRP as factors significantly correlated with clinical efficacy (*P* <.05). Receiver operating characteristic analysis confirmed that both renal function indicators (area under the curve = 0.848) and inflammatory markers (area under the curve = 0.739) served as reliable predictors of favorable treatment outcomes. The modified Yiqi Huoxue formula, as an adjunctive therapy, demonstrates significant clinical efficacy in the management of CGN. Its therapeutic benefits are potentially mediated through the dual mechanisms of improving renal function and attenuating systemic inflammation, thereby delaying disease progression. These findings support its clinical application for CGN patients.

## 1. Introduction

Chronic glomerulonephritis (CGN), commonly referred to as chronic nephritis, is clinically manifested by symptoms such as proteinuria, hematuria, edema, and hypertension, often accompanied by varying degrees of renal function impairment, severely threatening the patients’ safety.^[1]^ According to incomplete statistics,^[2]^ the incidence rate of chronic nephritis in China is around 10%, with primary glomerular diseases accounting for about 71%. In recent years, with the acceleration of the aging population, the incidence of chronic nephritis shows a rising trend annually. Therefore, adopting effective treatment measures is of significant importance to improve patients’ prognosis. Western medicine’s treatment methods for chronic nephritis mainly include corticosteroids, immunosuppressants, and blood pressure control drugs to delay disease progression.^[3]^ However, corticosteroids have notable side effects and are prone to relapse after discontinuation, failing to achieve the desired treatment effect.^[4]^ In recent years, traditional Chinese medicine (TCM) has demonstrated significant superiority in treating kidney diseases, providing a new approach for clinical treatment. In TCM, chronic nephritis falls under the categories of “edema,” “lumbago,” and “consumptive disease,” with the disease primarily located in the kidneys. The primary condition is kidney deficiency as kidneys are the foundation of congenital constitution, responsible for storing essence. When the kidneys are deficient, it impacts other organs, leading to essence and qi leakage, resulting in proteinuria.^[5]^

A summary and observation of outpatient medical records revealed that qi deficiency and blood stasis are the most common syndrome types in chronic glomerulonephritis. Based on clinical experience, the Yiqi Huoxue formula was formulated, this formula contains Astragalus, Angelica, and Earthworm, which have beneficial effects in tonifying Qi, promoting blood circulation, and alleviating pain by facilitating the flow of Qi and blood, thereby improving renal function. Codonopsis, Curcuma, and Achyranthes help invigorate blood, remove blood stasis, and enhance microcirculation, thus alleviating the damage caused by impaired renal blood flow. Corn silk, Plantago, Eclipta, and Arctium perform heat-clearing, diuretic, and detoxifying functions, effectively alleviating symptoms caused by edema while enhancing the elimination of metabolic waste products from the kidneys. These herbs work synergistically to address Qi deficiency, blood stasis, and renal dysfunction, providing a beneficial therapeutic effect on renal diseases induced by Qi deficiency and blood stasis.

This study, based on the pathogenic characteristics of chronic glomerulonephritis, applies the modified Yiqi Huoxue formula to treat chronic glomerulonephritis. Observing the changes in patients’ clinical symptoms, renal function indicators, and inflammatory factors before and after treatment, we aim to explore the clinical efficacy and safety of the Yiqi Huoxue formula, hoping to provide references for the clinical treatment of chronic nephritis.

## 2. Methods

### 2.1. Study participants

This study was approved by the Ethics Committee of Beijing Jingshun Hospital of TCM. The study subjects were selected from patients diagnosed with chronic glomerulonephritis who presented to the outpatient department of our hospital between April and August 2023. The inclusion criteria were as follows: All participants met the diagnostic criteria for chronic glomerulonephritis, as outlined in the sixth edition of the International Classification of Diseases (ICD-10), which included: The disease typically presents insidiously, with a slow progression that may vary in severity or alternatively manifest as a fluctuating pattern between mild and severe clinical manifestations. As the disease progresses, renal function gradually deteriorates, with later stages showing elevated serum creatinine, blood urea nitrogen (BUN), electrolyte imbalances, and anemia. Additionally, there is abnormal routine urinalysis with persistent and long-term proteinuria, generally not exceeding 3.5 g in a 24-hour urine protein quantification, as well as hematuria. These symptoms are accompanied by varying degrees of hypertension and edema. During the disease course, acute exacerbations acute nephritis-like episodes may be precipitated by infections, stress, or excessive fatigue. Once these factors have been excluded, a diagnosis of primary glomerulonephritis can be made; age 18 to 70 years; not having received related treatment within 1 month prior to enrollment; complete clinical data and awareness of the study content. The following individuals were excluded from the study: acute exacerbation of chronic nephritis or acute nephritis; severe cardiovascular and cerebrovascular diseases; severe liver and kidney dysfunction; chronic nephritis caused by secondary factors such as diabetes, systemic lupus erythematosus, drug-induced kidney damage; severe allergic constitution or allergy to the ingredients of the medications used in this study. Patients who met the inclusion criteria were randomly assigned to either the observation or control group.. This study was designed as a retrospective clinical study. All eligible patients who met the inclusion and exclusion criteria during the study period were consecutively enrolled. Due to the retrospective nature of the study and the lack of prior data, a formal sample size calculation was not performed. The final sample size of 90 cases was determined by the available medical record.

This study has been approved by the Ethics Committee of our hospital, and all patients signed an informed consent form. The study was conducted in accordance with the Declaration of Helsinki.

### 2.2. Treatment method

The control group was administered basic treatment and symptomatic support measures. Basic treatment measures included ample rest, avoidance of fatigue, and a diet low in salt and fat with high-quality protein. Symptomatic support included measures for lowering blood pressure and lipids. In the absence of contraindications, angiotensin-converting enzyme inhibitors or angiotensin II receptor blockers (ARB) were preferably administered to control blood pressure. In instances where urinary protein levels are below 1 g/24 hours, it is recommended that blood pressure be maintained at a level below 130/80 mm Hg. Conversely, when urinary protein levels are equal to or >1 g/24 hours, it is advised that blood pressure be controlled at a level below 125/75 mm Hg. For those who did not meet the blood pressure control standards, a combination of beta-blockers or calcium channel blockers could be used; for hyperlipidemia, statin drugs were the preferred choice.

The observation group, in addition to the treatment administered to the control group, was treated with a modified Qi-replenishing and blood-activating formula. The composition of the medication included: Astragalus 35 g, Codonopsis and Cornus fruit 20 g each, Atractylodes Macrocephala, Cicada molting, and Silkworm 15 g each, Dodder seeds, Goji berries, Cherokee Rose, Salvia, and Lycopus lucidus 10 g each. Medication modifications were made based on individual symptoms: for significant lumbar pain, Eucommia bark and Cibotium were added; for liver qi stagnation, Bupleurum and Trifoliate orange were added; for notable loss of appetite and abdominal bloating, Tangerine peel and Amomum were added; for yin deficiency, Glossy Privet fruit and Dried lotus grass were added; for damp-heat, Hedyotis and Common Selfheal fruit-spike were added; for notable hematuria, Notoginseng (taken as a powder) and Cattail pollen were added. Instructions for medication intake were: decoction of the herbs to obtain 300 mL of extract, advising patients to take it warm in 2 doses, morning and evening after meals. The decoction was provided by the hospital’s herbal medicine dispensary. The effectiveness indicators were evaluated for changes after 8 weeks of treatment in both groups.

### 2.3. Observation indicators

Clinical efficacy evaluation standards: In line with literature reports,^[6]^ evaluations are segmented into 4 tiers: clinical recovery, significantly effective, effective, and ineffective. Clinical recovery is evidenced when urine routine tests exhibit a negation of protein or a normalization of 24-hour urinary protein quantification; red cell count in urine routine tests is normalized or urine sediment red blood cell (RBC) count is normalized, and kidney function reverts to normal. Significantly effective is marked by a decrement of protein by 2 “+” in urine routine tests, or a reduction of ≥40% in 24-hour urinary protein quantification; RBC decrease by ≥3 per HP or 2 “+,” or urine sediment RBC count decreases by ≥40%, with kidney function being normal or essentially normal (not surpassing a 15% deviation from normal values). Effective is denoted by a decrement of protein by 1 “+” in urine routine tests, or a <40% reduction in 24-hour urinary protein quantification; RBC decrease by <3 per HP or 1 “+,” or urine sediment RBC count decreases by <40%; kidney function is normal or improved. Ineffective is characterized by no enhancement or deterioration in clinical symptoms and the aforementioned laboratory indicators (Table 1).

**Table 1:** Simplified clinical efficacy evaluation standards.

Evaluation category	Urine protein	RBC count	Kidney function
Clinical recovery	Normalization or negation	Normalization	Normal
Significantly effective	≥40% reduction or 2 + decrement	≥3 RBC decrease or ≥ 40% reduction	Normal or <15% deviation
Effective	1 + decrement or <40% reduction	<3 RBC decrease or <40% reduction	Normal or improved
Ineffective	No change	No change	No change

RBC = red blood cell.

Kidney function: pre and posttreatment, 5ml of fasting venous blood is procured from both groups, centrifuged to segregate the serum, and preserved at −80°Cfor analysis. An automatic biochemical analyzer is employed to assess serum creatinine (Scr) and BUN levels of both groups; all urine is collected from 8 am to 8 am the subsequent day, amalgamated, sampled, and a urine chemistry analyzer is utilized to evaluate the 24-hour urinary protein quantification (24hUPQ) level.

Lipid Indices: pre and posttreatment, an automatic biochemical analyzer is deployed to assess the levels of triglycerides (TG), total cholesterol (TC), and low-density lipoprotein cholesterol (LDL-C) in both groups.

Inflammation Indices: 5 mL of fasting venous blood is collected from both groups pre and posttreatment, centrifuged to segregate the serum, and preserved at −80°Cfor analysis. Enzyme-linked immunosorbent assay is utilized to evaluate the levels of serum interleukin-6 (IL-6), tumor necrosis factor-α (TNF-α), and high-sensitivity C-reactive protein (hs-CRP), strictly adhering to the assay kit instructions.

### 2.4. Statistical analysis

The data was processed using SPSS 23.0 software (SPSS Inc, Chicago). When the quantitative data followed a normal distribution, it was presented as mean ± standard deviation, and comparisons were made using the *t*-test. Categorical data was presented as (n [%]) and comparisons were made using the Chi-square test. A *P*-value of <.05 was considered statistically significant. To identify independent predictors of clinical efficacy, multivariate logistic regression analysis was performed. Variables that were significantly associated with clinical efficacy in univariate analysis were included in the logistic regression model. The odds ratio and 95% confidence intervals (CI) were calculated to evaluate the strength of the association between each predictor and clinical efficacy. Receiver operating characteristic (ROC) Analysis was used to assess the diagnostic performance of the selected predictors. Two separate models were constructed: Model 1: This model was built using the renal function-related indicators that showed a trend towards significance in univariate analysis. The predicted probabilities for each patient were calculated based on these renal function markers, which were then used to generate the ROC curve and calculate the area under the curve (AUC). Model 2: Similarly, Model 2 was constructed using the inflammatory biomarkers that exhibited a significant or trending association with clinical efficacy in univariate analysis. The predicted probabilities based on these inflammatory markers were used for ROC curve plotting and AUC calculation. The AUC values for both models were compared to determine their ability to distinguish between effective and ineffective treatments. An AUC value closer to 1.0 indicates better discriminatory power, while an AUC of 0.5 suggests no predictive ability.

## 3. Results

### 3.1. Baseline data

A total of 90 patients with chronic glomerulonephritis were included in this study, and according to the different treatment methods, they were divided into the observation group (n = 45) and the control group (n = 45). There was no statistically significant difference between the 2 groups in terms of gender, age, disease duration, and other baselin e data (*P* >.05), as shown in Table 2.

**Table 2:** Comparison of baseline data between the 2 groups.

Group	Observation group (n = 45)	Control group (n = 45)	*t*/χ^2^	*P*-value
Gender (n [%])				
Male	28 (62.22%)	31 (68.89%)	0.443	.506
Female	17 (37.78%)	14 (31.11%)		
Age (year)	50.78 ± 3.76	49.84 ± 4.52	1.072	.286
Disease duration (year)	4.98 ± 1.62	4.82 ± 1.47	0.491	.625

### 3.2. Clinical efficacy

The aggregate effectiveness rate in the observation group registered at 86.67%, markedly surpassing the 66.67% recorded in the control group. The divergence was statistically significant (*P* <.05), as delineated in Table 3.

**Table 3:** Comparison of clinical efficacy between the 2 groups.

Group	Observation group (n = 45)	Control group (n = 45)	χ^2^	*P*-value
Clinical cure	5 (11.11%)	3 (6.67%)	–	–
Significant effect	23 (51.11%)	8 (17.78%)	–	–
Effective	11 (24.44%)	19 (42.22%)	–	–
Ineffective	6 (13.33%)	15 (33.33%)	–	–
Total effectiveness rate (%)	86.67	66.67	5.031	.025

### 3.3. Renal function indicators before and after treatment

Prior to treatment, the comparative analysis of Scr, BUN, and 24hUPQ levels between the 2 groups exhibited no statistical significance (*P* >.05). Subsequent to treatment, the levels of Scr, BUN, and 24hUPQ in both groups manifested a decline compared to pretreatment levels (*P* <.05). Additionally, posttreatment levels of Scr, BUN, and 24hUPQ in the observation group were significantly subdued compared to those in the control group (*P* <.05), as explicated in Table 4.

**Table 4:** Comparison of renal function indicators before and after treatment between the 2 groups.

Group		Observation group (n = 45)	Control group (n = 45)	*T*	*P*-value
Scr (μmol/L)	Before treatment	110.84 ± 18.55	103.17 ± 23.92	1.7	.093
	After treatment	64.20 ± 7.09*	87.75 ± 8.60*	2.237	.028
BUN (mmol/L)	Before treatment	9.62 ± 1.35	9.47 ± 1.77	0.452	.652
	After treatment	6.82 ± 0.67*	8.41 ± 0.84*	9.927	<.001
24hUPQ (g/24h)	Before treatment	1.78 ± 0.24	1.74 ± 0.28	0.728	.469
	After treatment	0.65 ± 0.24*	1.00 ± 0.17*	7.983	<.001

24hUPQ = 24-hour urinary protein quantitation, BUN = blood urea nitrogen, Scr = serum creatinine.

* Compared with the same group before treatment, *P* <.05.

### 3.4. Blood lipid indices

Prior to treatment, the comparative analysis of TG, TC, and LDL-C levels between the 2 groups unveiled no statistically significant disparity (*P* >.05). Subsequent to treatment, the levels of TG, TC, and LDL-C in both groups exhibited a reduction compared to pretreatment levels (*P* <.05). The levels of TG, TC, and LDL-C in the observation group were significantly diminished compared to those in the control group posttreatment (*P* <.05), as elucidated in Table 5.

**Table 5:** Comparison of blood lipid indices before and after treatment between the 2 groups.

Group		Observation group (n = 45)	Control group (n = 45)	*t*	*P*-value
TG (mmol/L)	Before treatment	1.89 ± 0.50	1.90 ± 0.57	0.088	.93
	After treatment	1.02 ± 0.34*	1.38 ± 0.38*	4.736	<.001
TC (mmol/L)	Before treatment	5.66 ± 1.33	5.28 ± 1.20	1.423	.158
	After treatment	3.91 ± 0.54*	4.66 ± 0.75*	5.444	<.001
LDL-C (mmol/L)	Before treatment	3.75 ± 0.81	3.52 ± 1.07	1.15	.253
	After treatment	2.07 ± 0.61*	2.86 ± 0.87*	4.988	<.001

LDL-C = low-density lipoprotein cholesterol, TC = total cholesterol, TG = triglycerides.

* Compared to before treatment within the same group, *P* <.05.

### 3.5. Inflammatory indices

Prior to treatment, the levels of IL-6, TNF-α, and hs-CRP between the 2 groups exhibited no statistically significant disparity (*P* >.05). Subsequent to treatment, the levels of IL-6, TNF-α, and hs-CRP in both groups manifested a reduction compared to pretreatment levels (*P* <.05). The levels of IL-6 and hs-CRP in the observation group were significantly diminished compared to those in the control group posttreatment (*P* <.05), as elucidated in Table 6.

**Table 6:** Comparison of inflammatory indices before and after treatment between the 2 groups.

Group		Observation group (n = 45)	Control group (n = 45)	*t*	*P*-value
IL-6 (pg/mL)	Before treatment	18.75 ± 2.37	17.72 ± 2.81	1.88	.063
	After treatment	10.04 ± 2.19*	13.61 ± 2.02*	8.0.38	<.001
TNF-α (pg/mL)	Before treatment	51.99 ± 5.36	51.72 ± 6.38	0.217	.828
	After treatment	41.31 ± 3.23*	46.29 ± 2.82*	1.533	.129
hs-CRP (mg/L)	Before treatment	12.66 ± 2.40	12.20 ± 2.55	0.881	.381
	After treatment	7.81 ± 1.97*	9.80 ± 1.99*	4.767	<.001

hs=CRP = high-sensitivity C-reactive protein, IL-6 = interleukin-6, TNF-α = tumor necrosis factor-α.

* Compared to before treatment within the same group, *P* <.05.

### 3.6. Multivariate logistic regression analysis

The regression analysis results showed that the decrease in Scr levels was positively correlated with the improvement of treatment efficacy (OR = 1.75, 95% CI: 1.21–2.56, *P *= .004). Similarly, a decrease in BUN was significantly correlated with improvement in clinical efficacy (OR = 1.33, 95% CI: 1.02–1.73, *P *= .038). On the other hand, the reduction of 24hUPQ is also significantly associated with treatment efficacy (OR = 1.42, 95% CI: 1.07–1.88, *P *= .015). Although TG, TC, and LDL-C all showed significant changes after treatment, these lipid indicators did not show independent predictive effects on clinical efficacy in regression analysis (*P* >.05). In terms of inflammatory markers, the decrease of IL-6 is closely related to the improvement of clinical efficacy (OR = 1.89, 95% CI: 1.27–2.80, *P *= .002). The level of hs-CRP was also significantly correlated with treatment efficacy (OR = 1.54, 95% CI: 1.12–2.12, *P *= .009) (Table 7).

**Table 7:** Realistic regression analysis results.

Variable	OR (95% CI)	*P*-value
Scr	1.75 (1.21–2.56)	.004
BUN	1.33 (1.02–1.73)	.038
24hUPQ	1.42 (1.07–1.88)	.015
TG	1.05 (0.90–1.23)	.751
TC	1.04 (0.90–1.20)	.770
LDL-C	1.03 (0.90–1.18)	.805
IL-6	1.89 (1.27–2.80)	.002
hs-CRP	1.54 (1.12–2.12)	.009

24hUPQ = 24-hour urinary protein quantitation, BUN = blood urea nitrogen, CI = confidence interval, hs=CRP = high-sensitivity C-reactive protein, IL-6 = interleukin-6, LDL-C = low-density lipoprotein cholesterol, OR = odds ratio, Scr = serum creatinine, TC = total cholesterol, TG = triglycerides.

### 3.7. ROC analysis

**Figure 1.**
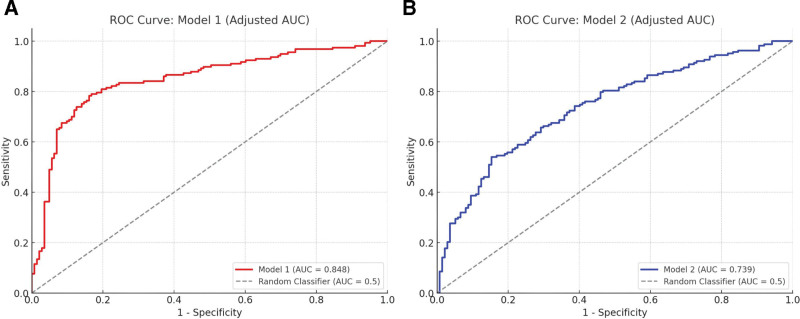
ROC curve and AUC comparison for Model 1 and Model 2. (A) (red curve) shows the ROC curve for Model 1, which includes the combination of Scr, BUN, and 24hUPQ, with an AUC of 0.848. (B) (blue curve) shows the ROC curve for Model 2, which includes the combination of IL-6 and hs-CRP, with an AUC of 0.739. The gray dashed line represents the random classifier (AUC = 0.5). 24hUPQ = 24-hour urinary protein quantitation, AUC = area under the curve, BUN = blood urea nitrogen, hs=CRP = high-sensitivity C-reactive protein, IL-6 = interleukin-6, ROC = receiver operating characteristic, Scr = serum creatinine.

To filter the regression analysis results into ROC analysis, we first performed ROC analysis using Model 1 that includes renal function indicators (Scr, BUN, 24hUPQ). The results showed that the model has high diagnostic ability in predicting clinical efficacy, with a comprehensive AUC of 0.848. When selecting the optimal cutoff value, the sensitivity was 84% and the specificity was 82%. Next, we constructed Model 2 and conducted ROC analysis on inflammatory markers (IL-6, hs-CRP). The results indicate that Model 2 has significant diagnostic ability in predicting treatment outcomes, with an AUC of 0.739. When selecting the optimal cutoff value, the sensitivity is 78% and the specificity is 74% (Fig. 1).

## 4. Discussion

According to incomplete statistics,^[7]^ the global prevalence of chronic kidney disease (CKD) is estimated to be between 8% and 16%, representing an increasingly severe public health issue worldwide. In China, the prevalence of CKD among the adult population is approximately 10.8%. Chronic glomerulonephritis is identified as the primary etiology of CKD, and it also stands as the leading primary disease leading to chronic renal failure.^[8]^ Over time, this condition can cause platelet aggregation within the glomerular capillaries, leading to coagulation, which in turn exacerbates renal function impairment. This sequence of events can trigger renal interstitial fibrosis and glomerulosclerosis, eventually progressing to renal failure^[9]^ with a poor prognosis. Therefore, timely control and treatment of the disease progression are crucial for improving patient outcomes. The exact cause of chronic glomerulonephritis remains unclear at this stage, and its pathogenesis is often associated with immunological and inflammatory factors.^[10]^ Clinically, there are no specific therapeutic drugs available. The primary goal of Western medicine treatment is to suppress proteinuria, protect renal function, and halt the deterioration of renal function. Commonly, angiotensin-converting enzyme inhibitors/ARB class drugs are used to dilate blood vessels, improve renal blood circulation, reduce high perfusion within the glomeruli, and protect renal function.^[11]^ TCM treatment, with its holistic approach, emphasizes syndrome differentiation and treatment. Based on the specific conditions of patients, it adopts individualized treatment plans. Owing to its low cost and minimal side effects, TCM is gradually manifesting significant therapeutic effects in the treatment of chronic glomerulonephritis.^[12]^

From the perspective of TCM, this prescription functions by tonifying Qi and promoting blood circulation. Modern pharmacological studies have shown that Astragalus and Pseudostellaria possess anti-inflammatory and immunomodulatory effects, which may contribute to the observed clinical benefits. Clinically, this treatment provides a complementary therapeutic option for patients who are intolerant or unresponsive to conventional therapies, and may help improve symptoms and quality of life.

In TCM, chronic nephritis is categorized under “edema,” “lumbar pain,” and “consumptive disease,” with the primary disease location being the kidneys. The fundamental deficiency is kidney deficiency since the kidney is considered the basis of congenital constitution, responsible for storing essence. When the kidney is deficient, it can affect other organs, leading to clinical manifestations like proteinuria due to the inability to retain essence and the spleen’s loss of control over essence and qi distribution. The spleen, regarded as the foundation of postnatal constitution, when deficient, lacks transformation and transportation abilities, thereby failing to control water, resulting in water and dampness stagnation, damp-heat accumulation in the lower energizer, and qi stagnation. This in turn affects water and fluid metabolism, leading to symptoms of edema. The occurrence and progression of this disease are related to spleen and kidney organ function abnormalities and the dysfunction of triple energizer qi transformation. The root of the disease lies in the kidney, with blood stasis and damp-heat as significant pathological products and causative factors, making them crucial elements in the disease progression. Therefore, treatment should follow the principles of replenishing the kidney, strengthening the spleen, promoting qi, activating blood circulation, and resolving dampness. The formula used in this study for promoting qi and activating blood circulation contains Astragalus, Codonopsis, and Atractylodes Macrocephala, all of which enter the spleen meridian and have the effect of replenishing qi and strengthening the spleen. Goji berries and Dodder seeds nourish the liver and kidneys, while Cherokee Rose and Cornelian Cherry fruit have the effect of securing kidney qi and preventing the leakage of essence. Salvia, Ze Lan (Lycopus lucidus), Cicada molting, and Silkworm help activate blood circulation and dispel stasis, and wind respectively. When used together, these herbs collectively replenish the kidney, strengthen the spleen, promote qi, activate blood circulation, expel wind, and resolve dampness.

Modern pharmacological studies have pointed out that the primary constituents in Astragalus, such as Astragalus polysaccharides and astragalosides, have renal protective effects.^[13]^ An animal study on Astragalus polysaccharides revealed^[14]^ that they could reduce the activity of IL-6 in the urine and serum of rats with mesangial proliferative glomerulonephritis, possibly by enhancing the body’s immune function, boosting macrophage phagocytosis of immune factors, reducing the deposition of immune complexes on the glomerular mesangium, and IL-6 secretion, thereby improving treatment efficacy. Codonopsis possesses bidirectional immune-regulating effects. Past research has indicated that Codonopsis peptides promote splenic lymphocyte proliferation through the Ca2+/CaN/NFATc1/IFN-γ signaling pathway, inducing Th1 and Th2 cell responses to secrete IFN-γ, TNF-α, and IL-10, thus enhancing human immunity.^[15]^ On the other hand, the ethyl acetate extract of Codonopsis can inhibit the expression and activity of pro-inflammatory cytokines IL-1β and IL-6 mRNA and the generation of reactive oxygen species, exerting anti-inflammatory effects through the PI3K/AKT pathway. Atractylodes Macrocephala, Goji berries, and Dodder seeds all exhibit notable anti-renal fibrosis, anti-inflammatory, lipid-lowering, and renal protective effects.

In this study, a modified Qi-replenishing and blood-activating formula was applied in the treatment of patients with chronic glomerulonephritis. The results demonstrated that the overall clinical effectiveness rate in the observation group was 86.67%, significantly higher than the 66.67% in the control group, with the difference being statistically significant. This indicates that the modified Qi-replenishing and blood-activating formula exhibited good clinical efficacy in treating chronic glomerulonephritis. Additionally, the study results showed that posttreatment levels of Scr, BUN, and 24hUPQ in the observation group were significantly lower than those in the control group, as were the levels of TG, TC, and LDL-C, with all differences being statistically significant. This suggests that the modified formula helps to improve renal function and lower lipid levels in patients with chronic glomerulonephritis, possibly due to its effects on improving renal microcirculation, modulating immunity, and enhancing hemorheology. The fundamental pathological characteristics of chronic nephritis include inflammatory responses, renal function impairment, and immune dysregulation, with IL-6 and hs-CRP being commonly used clinical indicators of inflammation.^[16,17]^ The study results revealed that posttreatment levels of IL-6 and hs-CRP in the observation group were significantly lower than those in the control group, with the difference being statistically significant, indicating that the modified formula helps to lower the levels of inflammatory markers in patients with chronic glomerulonephritis.

Multivariate logistic regression analysis identified that decreases in renal function markers, including Scr, BUN, and 24hUPQ, were strongly associated with improved clinical efficacy. This aligns with findings from studies such as Levin et al,^[18]^ which showed that markers of kidney function, including eGFR, improved the prediction of outcomes in CKD patients. In our study, the correlation of renal function indicators with treatment efficacy underscores the importance of monitoring renal function as a predictor for clinical outcomes in chronic glomerulonephritis. However, unlike studies like Gutiérrez et al,^[19]^ which highlighted that inflammatory biomarkers like TNFR-1 and KIM-1 could be associated with kidney failure progression, our findings suggest that lipid markers such as TG, TC, and LDL-C, although significantly altered by treatment, did not show independent predictive effects. This discrepancy may arise from differences in the clinical characteristics of the populations studied or the stage of kidney disease, highlighting the need for further investigation into the role of lipid metabolism in kidney disease.

In terms of ROC Analysis, Model 1, which combined renal function indicators, had an AUC of 0.848, suggesting high diagnostic ability in predicting treatment efficacy. This is consistent with findings from Keller et al,^[20]^ which demonstrated the association between cystatin C and kidney function markers in CKD. The high AUC observed in Model 1 supports the utility of renal biomarkers as reliable predictors of treatment outcomes. In contrast, Model 2, which included inflammatory markers (IL-6 and hs-CRP), had a lower AUC of 0.739, indicating that while inflammation plays a significant role in CKD progression, it is less predictive of clinical outcomes in this context compared to renal function markers. This is in line with the results of Petrović et al,^[21]^ who found that inflammatory markers are important but secondary to renal function markers in predicting CKD outcomes. Although inflammatory markers such as IL-6 and hs-CRP are crucial in understanding the pathophysiology of chronic glomerulonephritis, our findings suggest that the combined assessment of renal function provides more reliable prognostic information.

Both multivariate logistic regression analysis and ROC Analysis were conducted specifically for the treatment group receiving the modified Qi-replenishing and blood-activating formula. This approach allowed us to assess the independent predictive ability of renal function and inflammatory biomarkers in response to this treatment. By performing these analyses within the treatment group, we were able to gain insights into how the modified formula influences clinical outcomes, providing a more targeted evaluation of its efficacy in managing chronic glomerulonephritis.

This study has several limitations. First, it was conducted as a single-center study with a relatively small sample size, which may limit the generalizability of the findings. Second, while the analysis focused on short-term outcomes, long-term effects of the modified Qi-replenishing and blood-activating formula remain unclear and require further investigation. Third, we did not include a broader range of biomarkers or consider the impact of genetic factors, which may also influence treatment outcomes. Finally, the lack of a blinded design may introduce bias in the assessment of clinical efficacy, warranting future studies with more robust methodologies. This was a retrospective study with a relatively small sample size, and no prior sample size estimation was performed, which may limit the statistical power of the study and lead to potential overestimation of the treatment effect. Future prospective studies with larger sample sizes are warranted to confirm these findings.

## 5. Conclusion

In summary, the modified Qi-replenishing and blood-activating formula exhibited good clinical efficacy in treating chronic glomerulonephritis. It effectively lowered lipid and inflammatory marker levels, improved renal function indices, and delayed disease progression without adverse reactions, making it worthy of clinical promotion and application. Given its positive impact on both renal function and inflammation, this formula shows promise as a complementary or alternative treatment in the management of chronic glomerulonephritis, especially in patients with early-stage disease. Future multicenter, large-scale clinical trials and long-term follow-up studies are needed to further confirm its efficacy and safety profile, as well as its potential role in standard clinical practice.

## Author contributions

**Conceptualization:** Xi Liu, Jing Sun.

**Data curation:** Xi Liu, Jing Sun.

**Formal analysis:** Xi Liu, Jing Sun.

**Funding acquisition:** Xi Liu, Jing Sun.

**Investigation:** Xi Liu.

**Writing – original draft:** Xi Liu, Jing Sun.

**Writing – review & editing:** Xi Liu, Jing Sun.
